# A Case of Pneumocystis Pneumonia Developed During Rheumatoid Arthritis Treatment With Methotrexate and Golimumab

**DOI:** 10.7759/cureus.52944

**Published:** 2024-01-25

**Authors:** Toyoshi Yanagihara, Yusuke Oka, Atushi Moriwaki, Yuki Moriuchi, Hiroaki Ogata, Akiko Ishimatsu, Junji Otsuka, Kazuhito Taguchi, Makoto Yoshida

**Affiliations:** 1 Department of Respiratory Medicine, National Hospital Organization Fukuoka National Hospital, Fukuoka, JPN

**Keywords:** severe respiratory failure, methotrexate, golimumab, rheumatoid arthritis, pneumocystis jirovecii pneumonia

## Abstract

Here, we report a case of an 87-year-old female patient with rheumatoid arthritis (RA) treated with methotrexate (MTX) and golimumab who developed severe pneumocystis pneumonia (PCP), also known as *Pneumocystis jirovecii *pneumonia. The patient presented with chief complaints of dyspnea on exertion, dry cough, and fatigue. A high-resolution chest CT scan revealed diffuse, unevenly distributed ground-glass opacities throughout both lungs. The patient was clinically diagnosed with PCP based on the clinical settings, imaging, and a high level of serum β-D-glucan. While the patient required high-flow oxygen therapy, low-dose trimethoprim/sulfamethoxazole and corticosteroid therapy improved her condition, and the patient was discharged on day 25. Although to our knowledge no case report has been published regarding PCP in patients with RA treated with golimumab, this case emphasizes the importance of attention to opportunistic infections in elderly patients receiving immunosuppressive therapy. MTX use alongside tumor necrosis factor inhibitors like golimumab may increase the risk of serious infections such as PCP. The case underscores the necessity of prophylactic measures and early intervention for PCP, highlighting the delicate balance between immunosuppression benefits and infection risks in RA management.

## Introduction

The introduction of methotrexate (MTX) and biologic agents has revolutionized the treatment paradigm for rheumatoid arthritis (RA), markedly enhancing patient quality of life by managing symptoms and forestalling joint damage [1]. However, these therapies' immunosuppressive properties also heighten the risk of serious opportunistic infections. Pneumocystis pneumonia (PCP) is particularly concerning due to its potential for severe complications and a high mortality rate, with the risk further amplified in the elderly due to their declining immune function [2]. In this context, we present a case of an elderly patient with RA who developed PCP while undergoing treatment with MTX and the tumor necrosis factor (TNF) inhibitor, golimumab. This case underscores the need for vigilant monitoring and a proactive approach to managing the delicate balance between immunosuppression and infection risk in RA therapy.

## Case presentation

We report a case of an 87-year-old female patient with a long-standing history of RA. She was undergoing treatment with methotrexate 8 mg/week and monthly injections of the TNF inhibitor golimumab. The patient presented with chief complaints of dyspnea on exertion, dry cough, and fatigue. A month before admission, she started experiencing dry coughing. Three days before presenting to our facility, she experienced a worsening of exertional dyspnea and increased fatigue. Upon initial examination at a local clinic, her oxygen saturation (SpO_2_) was found to be 70%. A chest X-ray revealed bilateral pulmonary ground-glass opacities. Due to these findings, she was referred to our hospital for further evaluation and treatment.

**Figure 1:**
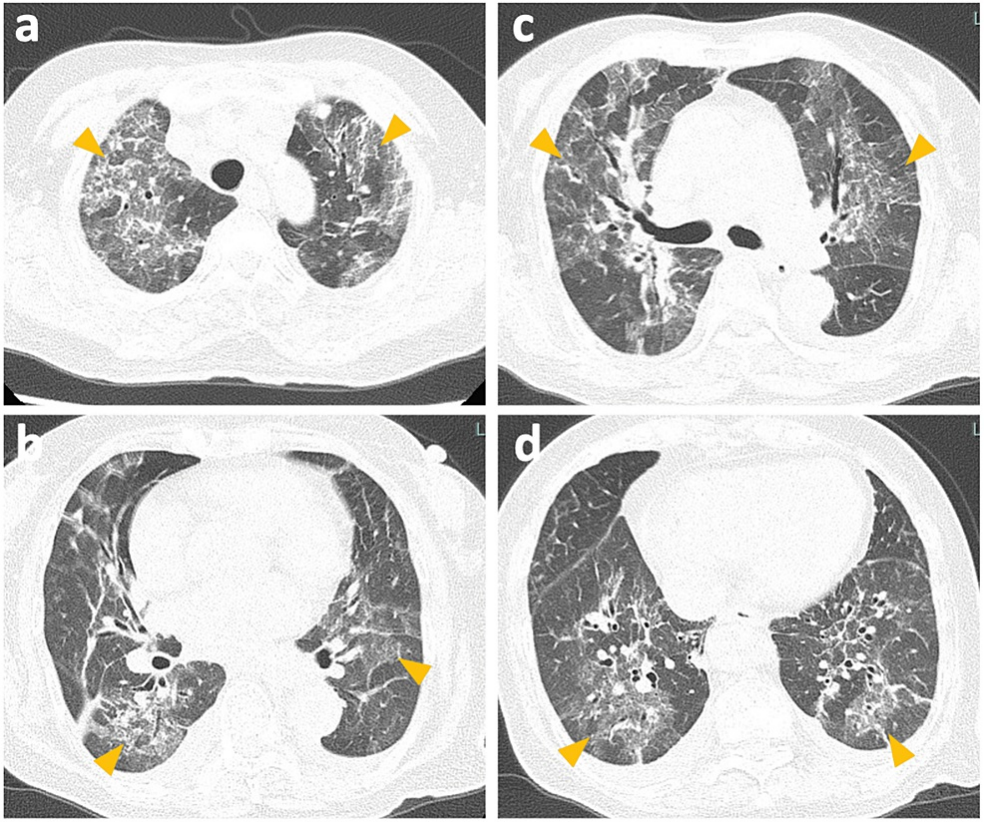
(a-d) Chest CT images of the patient on admission showing diffuse, unevenly distributed ground-glass opacities throughout both lungs (arrowheads)

Upon admission, her vital signs were as follows: pulse 75/min, blood pressure 137/72 mmHg, temperature 36.7°C, respiratory rate 20/min, and SpO_2_ 97% on 3 L/min of supplemental oxygen. Physical examination revealed inspiratory crackles in both lung fields, with no heart murmurs, edema, or joint pain. Her height was 146.0 cm, weight 45.1 kg, and BMI 21.16 kg/m². In rapid testing, the influenza antigen, SARS-CoV-2 antigen, urinary pneumococcal antigen, and urinary Legionella antigen were all negative. Laboratory findings revealed a WBC count of 13,380/uL, hemoglobin 9.5 g/dL, platelet count 344x10^3^/uL, CRP 20.8 mg/dL, lactate dehydrogenase level at 478 U/L, D-dimer at 6.6 µg/mL, and KL-6 397 U/mL (reference <500 U/mL). A high-resolution chest CT scan confirmed diffuse, unevenly distributed ground-glass opacities throughout both lungs (Figure 1).

**Figure 2:**
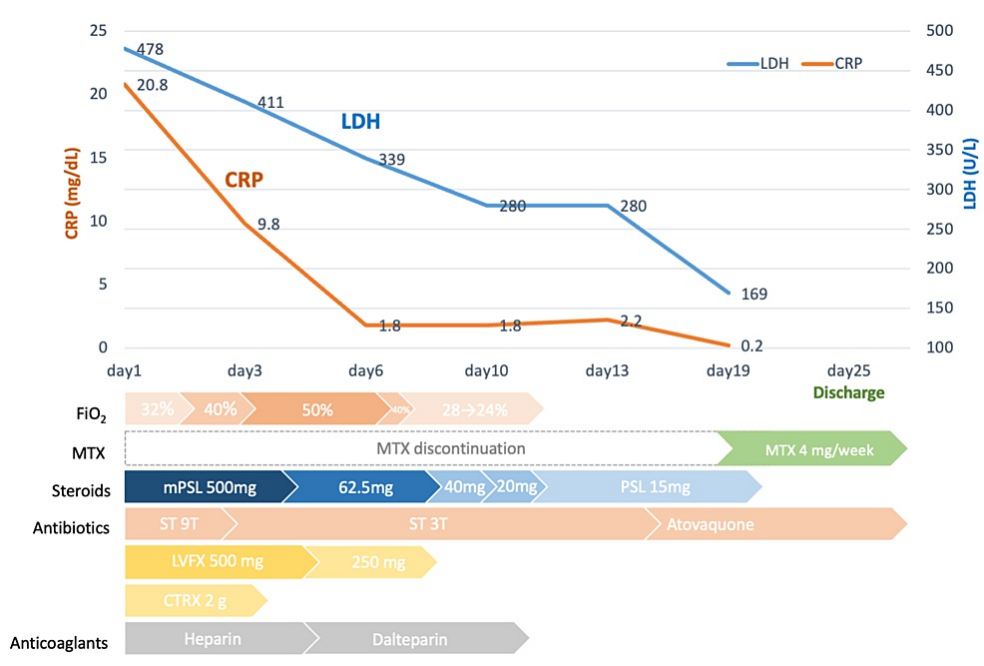
Clinical course of the patient MTX, methotrexate; mPSL, methylprednisolone; PSL, prednisolone; ST; trimethoprim/sulfamethoxazole; LVFX, levofloxacin; CTRX, ceftriaxone; LDH, lactate dehydrogenase; CRP, C-reactive protein

The differential diagnosis included RA-related interstitial lung disease, MTX-induced pneumonitis, and opportunistic infections such as pneumocystis pneumonia, cytomegalovirus pneumonia, and bacterial pneumonia. Treatment was initiated with discontinuation of MTX, administration of steroid pulse therapy (methylprednisolone, or mPSL, 500 mg), trimethoprim/sulfamethoxazole (TMP/SMX), antibiotics (levofloxacin 500 mg/day and ceftriaxone 2 g/day), and supplemental oxygen; 5000 units of Heparin Ca were administered due to elevated D-dimer levels (Figure 2).

On day 3, her respiratory status worsened, necessitating the initiation of high-flow oxygen therapy (Figure 2). On day 6, her serum β-D-glucan levels on admission were found to be elevated (423 pg/mL). Cytomegalovirus antigen on admission was negative. Considering her clinical background, progression, and imaging findings, she was clinically diagnosed with PCP. Treatment was continued with TMP/SMX (three tablets/day), steroids, and ongoing oxygen supplementation, and antibiotics were discontinued.

**Figure 3:**
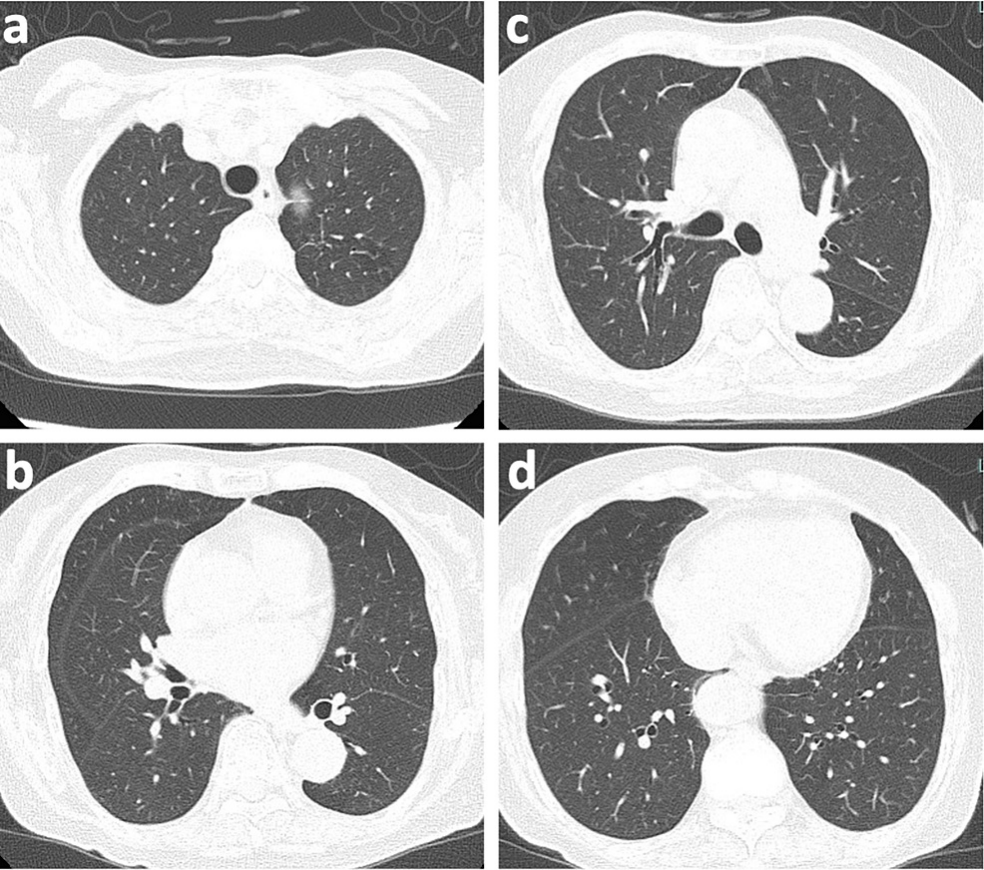
(a-d) Chest CT images of the patient on day 19 showing complete resolution of the ground-glass opacities

On day 12, the patient developed trunk pruritus and rash, prompting a transition from TMP/SMX to atovaquone. On day 19, her chest CT showed complete resolution of the ground-glass opacities (Figure 3), and blood tests indicated decreased inflammatory markers. Steroid treatment was discontinued, and MTX was resumed at a half dose of 4 mg, which had been paused. On day 25, she showed no recurrence of pneumonia and was discharged home in a stable condition.

## Discussion

In the GO-FORWARD study, a phase III double-masked controlled trial, the combination of golimumab with MTX was shown to enhance the therapeutic effect in RA when compared to MTX alone [3]. However, this combination also resulted in an increased rate of adverse events, including infections, though the specific breakdown of these infections was not detailed [3]. In Japan, additional post-marketing surveillance studies have shown a notable prevalence of PCP among RA patients treated with TNFα antagonists: infliximab (0.4% among 5000 patients), etanercept (0.2% among 7091 patients), and adalimumab (0.3% among 3000 patients) [4-6]. In a review article, the PCP incidence in patients with RA treated with golimumab was reported to be lower than that for other TNFα antagonists (0.03%; one case among patients) [7]. Nevertheless, to our knowledge, there is no case report regarding PCP in patients with RA treated with golimumab, which was a substantial motivation for reporting this case.

The risk factors for PCP are multifaceted and encompass age, gender, underlying pulmonary disease, and the use of immunosuppressive agents, including corticosteroids, biologic agents, and MTX. It is noteworthy that MTX is linked to a heightened risk of PCP, yet it simultaneously exhibits a reduced risk of other infections when contrasted with other RA treatment regimens, such as those involving corticosteroids [2]. Comparative case-control studies in RA patients treated with infliximab, a TNF inhibitor similar to golimumab, have identified several risk factors for PCP [8]. Being over the age of 65, taking more than 6 mg/day of prednisolone, and having pre-existing lung disease were all associated with an increased risk, with hazard ratios (HRs) of 3.77 (95% CI, 1.54-9.25), 3.76 (95% CI, 1.37-10.3), and 2.54 (95% CI, 1.00-6.46), respectively [8]. The presence of two or three of these risk factors significantly increases the incidence of PCP. In our case, the patient's advanced age was a prominent risk factor, which, combined with the use of MTX and golimumab, likely contributed to the development of PCP. These findings emphasize the necessity for heightened vigilance for PCP among older patients undergoing treatment with MTX and biologics for RA, particularly in the context of additional risk factors.

The patient was administered three tablets of TMP/SMX daily, delivering 240 mg of TMP (5.3 mg/kg/day), a dose lower than conventionally recommended. According to prior reviews and guidelines, the recommended first-line therapy for PCP is TMP/SMX at dosages ranging from 15 to 20 mg/kg/day of TMP [9-11]. Although the efficacy of TMP/SMX is established, it carries a significant risk of adverse events affecting the gastrointestinal, renal, hepatic, and hematologic systems. Nagai et al. recently assessed the efficacy and safety of low-dose TMP/SMX (TMP <12.5 mg/kg/day) for non-HIV PCP relative to conventional-dose TMP/SMX (TMP 12.5-20 mg/kg/day), accounting for patient demographic characteristics [12]. In the group receiving a lower dosage, the mean administration of TMP was 8.71 mg/kg/day, whereas it was 17.78 mg/kg/day in the group receiving the standard dosage. A comparison of 30-day mortality rates showed no marked difference (6.7% for the low-dose group versus 18.4% for the conventional-dose group; P = 0.080), even when adjusted for the characteristics of the patient population. Notably, the occurrence of adverse reactions, particularly nausea and hyponatremia, was appreciably reduced in the low-dose group (29.8% compared to 59.0% in the conventional-dose group; P = 0.005). Completion rates of initial treatment were 43.3% in the low-dose cohort and 29.6% in the conventional-dose cohort (P = 0.158). Consequently, a lower dosage of TMP/SMX correlated with fewer adverse reactions, maintaining comparable effectiveness in treating non-HIV PCP. Indeed, the patient's initial regimen of nine tablets of TMP/SMX led to nausea and abdominal discomfort, necessitating a dose reduction; the subsequent lower dose was successful in treating PCP.

The primary limitation of this case report is the absence of a definitive PCP diagnosis. The current definitive diagnosis for PCP relies on identifying trophic forms or cysts in bronchoalveolar lavage fluid through microscopic examination using specific stains like May-Grünwald-Giemsa, Gomori-Grocott, or immunofluorescence assay. Nonetheless, microscopic diagnosis can be challenging, especially with low fungal burdens, leading to potential false negatives. Moreover, the patient's advanced age and respiratory condition precluded bronchoscopy. PCP diagnosis was inferred from clinical history, imaging findings, and significantly elevated serum β-D-glucan levels. Serum β-D-glucan is a valuable marker when PCP is suspected, exhibiting a sensitivity of 92% and a specificity of 86% at a cutoff of 31 pg/mL, resulting in a positive likelihood ratio of 6.57 [13]. With such a high post-test probability, PCP was the clinical diagnosis. The absence of any interstitial lung abnormalities in subsequent CT scans, alongside a successful MTX rechallenge, further reduced the likelihood of MTX-induced pneumonitis or exacerbation of RA-associated interstitial lung diseases, initially considered in the differential diagnoses.

## Conclusions

This case illuminates the critical balance in RA treatment between managing disease activity and mitigating infection risk, especially in elderly patients on immunosuppressive therapy. It serves as a poignant reminder of the importance of considering prophylactic measures against PCP when using MTX and biologics such as golimumab. Clinicians must maintain a high index of suspicion for opportunistic infections like PCP, to ensure prompt diagnosis and treatment when it comes to this vulnerable population.
